# Diabetes Mellitus Is Associated with Distinctive Aortic Wall Degeneration During Acute Type A Aortic Dissection

**DOI:** 10.3390/jcm14134731

**Published:** 2025-07-04

**Authors:** Santtu Heikurinen, Ivana Kholova, Timo Paavonen, Ari Mennander

**Affiliations:** 1 Department of Cardiothoracic Surgery, Tampere University Hospital, Heart Hospital, 33520 Tampere, Finland; santtu.heikurinen@tuni.fi; 2 Faculty of Medicine and Health Technology, Tampere University, 33520 Tampere, Finland; ivana.kholova@tuni.fi (I.K.); timo.paavonen@tuni.fi (T.P.); 3 Department of Pathology, Fimlab Laboratories, 33520 Tampere, Finland; 4 Institute of Clinical Medicine, Pathology and Forensic Medicine, University of Eastern Finland, 70210 Kuopio, Finland; 5 Department of Clinical Pathology, Diagnostic Imaging Center, Kuopio University Hospital, 70029 Kuopio, Finland

**Keywords:** aortic wall atherosclerosis, acute type A aortic dissection, diabetes mellitus

## Abstract

**Background:** Non-adjustable patient characteristics such as diabetes mellitus may influence surgical decision-making and outcome after acute type A aortic dissection (ATAAD). The aim of this study was to compare the degree of aortic wall atherosclerosis and surgical solutions in patients with diabetes mellitus versus those without during ATAAD. **Methods:** Altogether, 123 consecutive patients undergoing surgery for ATAAD at Tampere University Heart Hospital were evaluated. The ascending aortic wall resected in surgery was processed for histopathological analysis of atherosclerosis, inflammation, and medial layer degeneration. Patients with and without diabetes mellitus were compared during a mean 4.7-year follow-up. **Results:** There were 11 patients with diabetes mellitus and 112 without. The mean age for all patients was 63.6 years (standard deviation [SD] 13.3). Altogether, 48 patients had a conduit aortic prosthesis replacing the aortic root together with the ascending aorta, including only one patient with diabetes (*p* = 0.049). Nine patients received a frozen elephant trunk prosthesis to treat the aortic arch together with the ascending aorta. The severity of ascending aorta atherosclerosis was more prominent in patients with diabetes mellitus as compared to patients without (0.8 [0.4] vs. 0.3 [0.5], *p* = 0.009, respectively). During follow-up, 8 and 78 patients with and without diabetes died, respectively (logarithmic rank *p* = 0.187). **Conclusions:** Histopathology of the ascending aorta during ATAAD reveals distinctive severity of aortic wall atherosclerosis in patients with diabetes mellitus versus those without. The degree of atherosclerosis assessed postoperatively is associated with the extent of surgical procedure in many patients and may guide follow-up protocol.

## 1. Introduction

Some non-adjustable patient characteristics may add to increased risk after surgery for acute type A ascending aorta dissection (ATAAD) [1,2]. While ATAAD is an emergency challenge, the primary aim is to save the patient during surgery [3]. The initial surgical technique, including resection of the primary intimal tear and the estimation of disease extension, determines the surgical complexity. Upon successful surgical treatment for ATAAD, non-adjustable patient characteristics such as diabetes mellitus may further influence outcome [4].

Diabetes mellitus is a growing and chronic systematic disease affecting 10.5% of the global population [4]. Diabetes mellitus is associated with increased arteriosclerosis and stiffness of the arterial circulation [5]. Few studies have investigated the association between diabetes mellitus or hyperglycemia and ATAAD [6,7]. Some studies have even shown that diabetes mellitus as a cardiovascular risk factor does not increase early mortality in patients with ATAAD as compared to those without diabetes mellitus [8,9]. Nonetheless, only a few researchers have studied the association between aortic wall histopathology and outcome after life-saving surgery [10,11,12].

Since the primary intimal tear often occurs in the ascending aorta during ATAAD, a limited surgery including simple supracoronary reconstruction of the ascending aorta is considered suitable for many; the extension of the disease proximally encompassing the aortic root requires a conduit prosthesis or a supracoronary prosthesis [1,3]. The extent of surgical extension depends on if a reconstructive surgical approach is feasible including the ascending aorta with or without the aortic root. The primary intimal tear often needs resection, and the aortic layers need to be re-approximated proximally to prevent further blood inflow into the false lumen [13]. However, the characteristics and extension of ATAAD in patients with diabetes remain controversial.

We hypothesize that the extension of ATAAD in patients with diabetes mellitus is associated with ascending aortic wall histological changes; the ascending aortic wall arteriosclerosis would be more prominent in patients with diabetes. The objective of this study was to evaluate the plausible association of aortic wall atherosclerosis and the extent of proximal aortic disease in patients undergoing ATAAD with diabetes mellitus versus those without.

## 2. Materials and Methods

### 2.1. Ethical Statement and Study Design

After approval by the institutional review board (Ethical Committee of the Tampere University Hospital, Tampere, Finland, R13077 and R23028 on 29 April 2019 and 20 May 2013, respectively), the need for informed consent was waived. This study was conducted according to the Declaration of Helsinki (as revised in 2013). The authors are accountable for all aspects of this study; questions related to the accuracy or integrity of any part of the work are appropriately investigated and resolved. Acute type A aortic dissection was preoperatively confirmed using computer tomography (CT) and echocardiography, whenever possible [14]. The largest diameter of the ascending aorta was retrospectively measured from CT images. The severity of aortic valve regurgitation included moderate to severe regurgitation graded as 2–3 out of 3. Surgeries were performed between January 2006 and November 2019. Patients with connective tissue disorders including Marfan, Turner, and Loeys–Dietz syndromes were excluded (*n* = 3).

### 2.2. Surgery

The decision on the extension of the resection and surgical technique during ATAAD was at the discretion of the operating surgeon. If hypothermic circulatory arrest was employed, the core temperature was set at 20–24 °C. If the aortic wall, including the sinotubular junction (STJ), was estimated as the reason for aortic regurgitation, the STJ was tailored for a suitable graft in an aortic root-sparing fashion with/without aortic valve surgery [15]. Whenever ATAAD included the root of the aorta, radical resection of the dissecting and dilated ascending aorta was performed, including the root and the aortic valve. The aortic arch was resected completely or in a hemiarch fashion depending on the involvement of aortic wall disease. The intimal tears were resected whenever possible. The size of the graft was estimated by the principal surgeon. Since the surgical procedure was performed upon surgical decision, the sample was procured from the resected area of the ascending aorta in the vicinity of the STJ.

### 2.3. Histopathology and Immunohistochemistry

A minimum of six pieces of resected ascending aorta including all three aortic wall layers, i.e., the intima, the media, and the adventitia, were embedded in paraffin, cut to 4 m thick sections and stained with hematoxylin and eosin, Alcian Blue and periodic acid–Schiff, and Verhoeff–van Gieson. At least 18 sections (6 stained with hematoxylin–eosin, 6 stained with Verhoeff–van Gieson and 6 with Alcian Blue and periodic acid–Schiff) were evaluated in each case.

Hematoxylin–eosin was used for the overall investigation of degeneration, aortic wall inflammation and presence of atherosclerosis. Verhoeff–van Gieson staining was applied in the assessment of elastic fibers including laminal medial collapse. Alcian Blue and periodic acid–Schiff was used for mucoid extracellular matrix accumulation assessment and fibrosis evaluation. The presence of ascending aorta overall medial degeneration and atherosclerosis were categorized as missing, mild, moderate or severe. The pattern of ascending aorta inflammation was defined as non-existent, mild, moderate or severe. The aortic specimens were assessed as a part of routine surgical pathology evaluation according to the guidelines of The Society for Cardiovascular Pathology and The Association for European Cardiovascular Pathology [16,17] by two experienced cardiovascular pathologists (I.K., T.P.). The worst present grade/distribution was reported and semi-quantified on a scale of 0–3 [16,17].

### 2.4. Follow-Up Protocol

Documentation of mortality and morbidity was available for all patients. Follow-up consisted of physical examination, CT and echocardiography three months after surgery, and yearly CT thereafter. The mean follow-up for the patients was 4.7 years (standard deviation [SD] 0.3). Morbidity after surgery included aortic reoperation because of reverse aortic remodeling that included the need for proximal or distal aortic reoperation with an onset of new aortic dissection or rupture, or evidence of increased aortic aneurysm observed by echocardiography or CT. According to our institutional policy, the aortic aneurysm included an aortic diameter greater than 5.0 cm or aortic growth greater than 1 cm in a year.

### 2.5. Statistical Analysis

Continuous variables were expressed as mean values with SD and compared using the Mann–Whitney U test. Categorical variables were presented as numbers and percentages and were compared using χ^2^ or Fisher’s exact tests. The patients were divided into two groups according to the presence of preoperative diabetes mellitus without detailed information on glycemic control and mean glycated hemoglobin. Unadjusted survival was evaluated using Kaplan–Meier analysis with logarithmic rank tests. Multivariable Cox regression analysis was performed to analyze the association of diabetes with survival adjusted for clinically relevant confounders. These confounders included the presence of atherosclerosis, male sex, age, hypertension, smoking, cardiovascular shock, pericardial tamponade, previous cardiac intervention, and presence of primary ascending aorta tear. All analyses were performed with IBM SPSS Statistics version 28.0 (IBM Corporation, Armonk, NY, USA) with *p* < 0.05 as the significance criterion. Graphs were created using R version 4.4.0 (R Foundation for Statistical Computing, Vienna, Austria) and RStudio version 2024.09.0 + 375 (RStudio, PBC, Boston, MA, USA) utilizing the survival and survminer packages.

## 3. Results

### 3.1. Patient Characteristics

This study included 123 consecutive patients without Marfan syndrome or any other syndrome who underwent ATAAD surgery and whose surgical specimens were processed for histology. All patients experienced the onset of symptoms leading to surgery for ATAAD in less than 2 weeks. Patient characteristics are shown in Table 1.

There were 11 patients with diabetes mellitus and 112 without. Altogether, there were 78 male patients (63.4%). The mean age for all patients was 63.6 years (SD 13.3). There were no statistical differences among the patients with or without diabetes mellitus in body mass index, ejection fraction, creatinine, and lactate levels (*p* = 0.402, *p* = 0.531, *p* = 0.847, *p* = 0.105, respectively). Euroscore II was 29.5 (SD 27.7) and 20.3 (SD 16.7) in patients with and without diabetes mellitus (respectively, *p* = 0.559). A total of 86 patients had hypertension, there were 20 active smokers, preoperative shock occurred in 39 patients, and pericardial tamponade in 43 patients. Seventeen patients had a previous cardiac surgery and ten patients had a previous percutaneous coronary intervention. Ten patients had a bicuspid aortic valve, all without diabetes mellitus.

### 3.2. Description of ATAAD

The ascending aortic diameter was 58.3 mm (22.5) in patients with diabetes mellitus and 53.5 mm (11.0) in those without (*p* = 0.718). Altogether, a primary intimal tear of the ascending aorta was found in 53 (43.1%) patients. The tear extended into the aortic root in 59 (48.0%) and included the aortic arch in 4 patients. The primary intimal tear was undetected in seven patients, all without diabetes mellitus. Coronary, cerebral, spinal renal, visceral, and limb malperfusions were equally distributed among the patients.

### 3.3. Operative Techniques

The operative techniques are shown in Table 2.

Most of the patients (*n* = 75, 61.0%) had a replacement of the ascending aorta with a tube prosthesis including a proximal anastomose in the STJ and a distal anastomose in a hemiarch fashion of the lesser curvature of the aortic arch. Of these, only three patients also had a separate aortic valve replacement due to aortic valve regurgitation. In addition, 48 patients had a conduit aortic prosthesis replacing the aortic root together with the ascending aorta, including only one patient with diabetes (*p* = 0.049). Nine patients received a frozen elephant trunk prosthesis to treat the aortic arch together with the ascending aorta. Hypothermic circulatory arrest was used in most cases (*n* = 109, 92.4%). Concomitant coronary artery bypass grafting was performed in 22 patients.

### 3.4. Postoperative Findings

**Figure 1:**
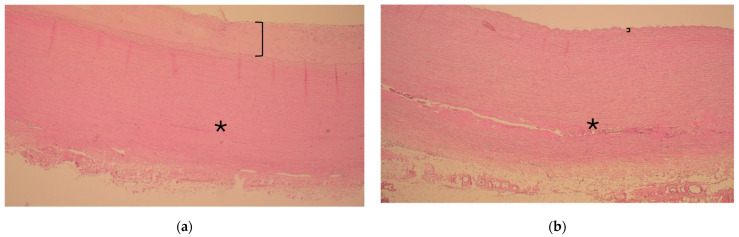
Representative histology of ascending aorta in the vicinity of the sinotubular junction during acute type A aortic dissection in a patient with and without diabetes ((**a**) and (**b**), respectively). Note the thickness of the intima layer (bracket) representing atherosclerosis and consistent media layer without extension of dissection (asterisk *) in (**a**). In contrast, note the clear continuation of the dissection tear inside the media layer (asterisk *) and slim intima layer without atherosclerosis (bracket) in (**b**). Hematoxylin and eosin, 40× magnification.

**Figure 2:**
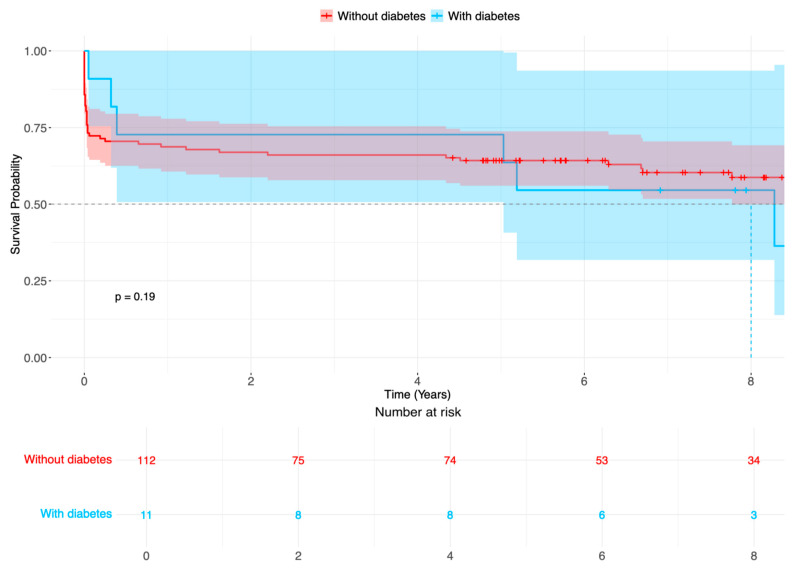
Survival probability (%) of patients after surgery for acute aortic type A dissection in patients with diabetes (blue line) compared with patients without diabetes (red line). Time-varying outcome according to Kaplan–Meier estimation. Log rank *p* = 0.187.

There were no major differences in the histopathology of the aortic wall in patients with or without diabetes as the resected ascending aorta had experienced degeneration but seldom inflammation. However, ascending aorta atherosclerosis was more severe in patients with diabetes mellitus as compared to those without (0.8 vs. 0.3, respectively, *p* = 0.009), as shown in Table 3 and Figure 1 and Figure 2.

### 3.5. Aortic Reoperations and Survival

Patient outcomes are shown in Table 4.

There were no statistical differences in outcome variables; altogether, strokes occurred in 38 patients (30.9%), 16 patients (13.0%) required dialysis after surgery for ATAAD, 16 patients had respiratory insufficiency, and there were 5 patients with heart failure. Reintervention included eight aortic root reinterventions, none of which had diabetes mellitus. The early 30-day mortality occurred in 17 patients (13.8%). During follow-up, 8 and 78 patients with and without diabetes died, respectively (Figure 2, logarithmic rank *p* = 0.187).

According to the adjusted multivariable Cox regression analysis, the presence of diabetes mellitus (hazard ratio [HR]: 0.69, 95% confidence interval [CI]: 0.21–2.23, *p* = 0.537) was not a significant factor related to all-cause mortality. Instead, age and high preoperative lactate levels were risk factors for all-cause mortality (HR: 1.05, 95% CI: 1.01–1.09, *p* = 0.019 and HR: 1.31, 95% CI: 1.14–1.50, *p* < 0.001, respectively; Table 5).

## 4. Discussion

This study shows that the severity of ascending aorta atherosclerosis during ATAAD is increased in patients with diabetes as compared with those without. A more limited surgical solution including a supracoronary aortic prosthesis may well be feasible during ATAAD in many patients with diabetes.

Despite the presence of diabetes mellitus, other clinical patient characteristics are mainly similar among the groups [18,19]. Patient presentation includes ATAAD with various combinations of malperfusion symptoms, occasional aortic valve regurgitation, and the presence of a primary intimal tear [20]. The primary intimal tear is often found in the ascending aorta, and the dissection may extend proximally to the aortic root or distally beyond the aortic arch [20]. However, a conduit prosthesis was only required in one patient with diabetes. As compared with patients without diabetes mellitus, a stiff atherosclerotic ascending aorta suggested use of a simple tube graft solution in patients with diabetes mellitus. In the acute setting of ATAAD, diabetes mellitus may not add to poor outcome after surgery [6].

According to Cox multivariable proportional hazards regression analysis of perioperative variables potentially associated with all-cause mortality among the patients with and without diabetes, diabetes mellitus did not increase the risk for all-cause mortality. The follow-up outcome was similar in all the patients after either a conduit prosthesis replacing the aortic root together with the ascending aorta or a root-sparing aortic prosthesis replacing only the ascending aorta. On the other hand, only a minority of the patients had aortic arch surgery including a frozen elephant trunk prosthesis, while extension of ATAAD encompassed the aortic arch distally beyond zone 2. Reintervention was relatively seldom needed; a conservative approach seems justified.

During surgery for ATAAD, the aortic root is replaced if there is an intimal flap in the sinus of Valsalva, severe aortic valve regurgitation is present, and the coronary artery ostia are involved [15,20]. Circulatory malperfusion, pericardial tamponade, strokes, and comorbidities also affect surgical decision-making; surgery for the aortic root is also generally recommended during annuloaortic ectasia [2,19]. Estimation of aortic wall tissue frailty is thought to aid in the surgical decision-making of the extension of aortic resection [15]. This study shows that decision-making regarding aortic root resection was often consistent with the postoperative finding of atherosclerosis of the ascending aorta.

Recently, ATAAD was histopathologically reported as encompassing weaknesses in the congenital versus acquired aortic wall [21] or including mixed versus degenerative compounds [8]. The nomenclature and definitions seem to overlap, as many degenerative aortic walls were described as being without atherosclerosis compared to mixed aortic walls with atherosclerosis and degeneration [10]. Previous studies have seldom discussed the extent of the histopathological findings versus surgical techniques used during ATAAD [22], but the involvement of the aortic root disease phenotype of ATAAD seems to be more prevalent during the congenital and degenerative forms of aortic wall weaknesses.

The association of diabetes mellitus with an increase in arteriosclerosis may well indicate aortic wall atherosclerosis. Though atherosclerosis per se may add to disease outcome in general, aortic wall atherosclerosis may provide aortic stability during ATAAD [18,23]. Instead, the severity of aortic wall weakness may be associated with medial wall degeneration observed as mucoid extracellular matrix accumulation, a histopathological marker justifying root replacement in a previous study [24,25]. Insulin resistance [12] and stress-induced hyperglycemia [7] themselves may add to the phenotypic switch of vascular smooth muscle cells and the vulnerability of patients with ATAAD, respectively. In contrast, the degree of ascending aortic wall medial degeneration per se did not differ in our patients with or without chronic diabetes mellitus.

During the mid-term follow-up of our patients after surgery for ATAAD, diabetes mellitus is not an independent variable for all-cause mortality when other non-adjustable confounders or clinical variables, i.e., age, sex, hypertension, smoking, presence of shock, tamponade, location of primary intimal tear, malperfusion, and the presence of bicuspid aortic valve are considered [26]. The message is in line with Lorenz et al., who showed that early outcome after surgery for ATAAD is not associated with the presence of diabetes mellitus [6]. Instead, long-term mortality after ATAAD in patients with diabetes mellitus may be due to complications associated with diabetes during follow-up rather than diabetes mellitus per se [6]. The location of the intimal tear, moderate to severe aortic valve regurgitation, male sex, age, the presence of a bicuspid aortic valve, and increased aortic diameter are all part of the decision-making for the extent of surgery encompassing the surgical decision-making in any patient [1]. If the aortic root appears not to be involved during ATAAD, careful inspection of the quality of aortic tissue is routine during surgery, and the decision to replace the aortic root is performed according to clinical judgment to ensure blood circulation in the true lumen. It is tempting to suggest that decision-making on the extent of surgery during ATAAD may be pondered similarly in patients with or without diabetes mellitus.

This pilot study represents a contemporary single-center cohort from real life. Larger multicenter studies are warranted to confirm the results of this study. The limitations of this study include the small number of patients and the relatively short follow-up; aortic wall histology is obviously only available in patients who underwent surgery. Histology was available for almost 85% of the patients. Aortic root surgery together with the ascending aorta for ATAAD depends on the discretion of the surgeon. Different medication protocols and types of diabetes mellitus are not identified. Insulin resistance, glycemic control such as the proportion of glycosylated hemoglobin abnormalities or time since diabetes diagnosis were not assessed. Postmortem investigation data are not available. Despite many entry tear locations being difficult to confirm once the ascending aorta is transected during ATAAD, there are only, remarkably, seven (5.7%) unreported or unknown sites of entry tears. There is a need to further study detailed morphological evidence of aortic wall changes during ATAAD in patients with diabetes mellitus.

## 5. Conclusions

Distinctive histopathological features of aortic wall degeneration were present during ATAAD in patients with diabetes mellitus versus without. Though a conservative aortic root-sparing surgery was offered to all but one patient with diabetes mellitus, postoperative outcome and survival did not differ among any of the patients. It remains to be confirmed whether the presence of ascending aorta atherosclerosis may be associated with a resistant aortic root against proximal extension of ATAAD.

## Figures and Tables

**Table 1:** Patient characteristics.

Characteristics	All Patients	With Diabetes	Without Diabetes	*p*-Value
Number of patients, *n*	123	11	112	
Age, years (SD)	63.6 (13.3)	69.7 (9.1)	63.0 (13.5)	0.099
Male, *n*	78 (63.4%)	5 (45.5%)	73 (65.2%)	0.208
BMI	27.9 (4.8)	28.9 (3.2)	27.8 (4.9)	0.402
Hypertension, *n*	86 (69.9%)	9 (81.8%)	77 (68.8%)	0.502
COPD, *n*	9 (7.3%)	0	9 (8.0%)	>0.99
Smoking, *n*	20 (16.3%)	3 (27.3%)	17 (15.2%)	0.384
Shock, *n*	39 (31.7%)	5 (45.5%)	34 (30.4%)	0.323
Previous aortic dilatation, *n*	32 (26.0%)	1 (9.1%)	31 (27.7%)	0.285
Previous cardiac surgery, *n*	17 (13.8%)	3 (27.3%)	14 (12.5%)	0.178
Previous percutaneous intervention, *n*	10 (8.1%)	1 (9.1%)	9 (8.0%)	>0.99
Salvage, *n*	8 (6.5%)	0	8 (7.1%)	>0.99
Tamponade, *n*	43 (35%)	5 (45.5%)	38 (33.9%)	0.513
Malperfusion, *n*	57 (46.3%)	7 (63.6%)	50 (44.6%)	0.343
Coronary malperfusion, *n*	20 (16.3%)	2 (18.2%)	18 (16.1%)	>0.99
Cerebral malperfusion, *n*	13 (10.6%)	2 (18.2%)	11 (9.8%)	0.328
Spinal malperfusion, *n*	4 (3.3%)	0	4 (3.6%)	>0.99
Renal malperfusion, *n*	41 (33.3%)	6 (54.5%)	35 (31.3%)	0.177
Visceral malperfusion, *n*	11 (8.9%)	1 (9.1%)	10 (8.9%)	>0.99
Limb malperfusion, *n*	23 (18.7%)	2 (18.2%)	21 (18.8%)	>0.99
Ascending aorta tear, *n*	53 (43.1%)	6 (54.5%)	47 (42.0%)	0.528
Aortic root tear, *n*	59 (48.0%)	5 (45.5%)	54 (48.2%)	>0.99
Aortic arch tear, *n*	4 (3.3%)	0	4 (3.6%)	>0.99
Descending aorta tear, *n*	1 (0.8%)	0	1 (0.9%)	>0.99
Unknown tear location, *n*	7 (5.7%)	0	7 (6.3%)	>0.99
Aortic diameter, mm (SD)	53.9 (11.8)	58.3 (22.5)	53.5 (11.0)	0.718
AR, *n*	74 (60.2%)	7 (63.6%)	67 (59.8%)	>0.99
BAV, *n*	10 (8.1%)	0	10 (8.9%)	0.597
Creatinine	92.5 (30.0)	90.6 (29.1)	92.7 (30.2)	0.847
Lactate	3.3 (3.4)	4.3 (3.6)	3.2 (3.4)	0.105
Ejection fraction, %	54 (50)	45 (52)	55 (50)	0.531
Euroscore II	20.9 (17.5)	29.5 (27.7)	20.3 (16.7)	0.559

BMI = body mass index; COPD = chronic obstructive pulmonary disease; CAD = coronary artery disease; CABG = coronary artery bypass graft surgery; BAV = bicuspid aortic valve; AR = moderate to severe aortic valve regurgitation; SD = standard deviation.

**Table 2:** Operative details.

Details	All Patients	With Diabetes	Without Diabetes	*p*-Value
Number of patients, *n*	123	11	112	
Conduit operation, *n*	48 (39.0%)	1 (9.1%)	47 (42.0%)	0.049
Mechanical conduit, *n*	25 (20.3%)	0	25 (22.3%)	0.118
Biological conduit, *n*	23 (18.7%)	1 (9.1%)	22 (19.6%)	0.688
Graft replacement of ascending aorta, *n*	75 (61.0%)	10 (90.9%)	65 * (58.0%)	0.049
Mechanical valve + supracoronary prosthesis, *n*	2 (1.6%)	0	2 (1.8%)	>0.99
Biological valve + supracoronary prosthesis, *n*	1 (0.8%)	0	1 (0.9%)	>0.99
Frozen elephant trunk, *n*	9 (7.3%)	2 (18.2%)	7 (6.3%)	0.185
Concomitant surgery, *n*	24 (19.5%)	1 (9.1%)	23 (20.5%)	0.690
CABG, *n*	22 (17.9%)	1 (9.1%)	21 (18.8%)	0.687
Mitral surgery, *n*	1 (0.8%)	0	1 (0.9%)	>0.99
Hypothermic circulatory arrest, *n*	109 (92.4%)	9 (90.0%)	100 (92.6%)	0.563
Hypothermic circulatory arrest time, min (SD)	12.5 (20.4)	8.3 (9.6)	12.8 (21.0)	0.712
Aortic cross clamp time, min (SD)	180.6 (91.1)	137.6 (51.0)	185.0 (93.3)	0.128
Cardiopulmonary bypass time, min (SD)	256.8 (121.6)	218.4 (117.5)	259.9 (122.1)	0.413

* One patient without diabetes died before any graft replacement of ascending aorta could be attempted.

**Table 3:** Histopathological evaluation and assessment.

Variables		All Patients	With Diabetes	Without Diabetes	*p*-Value
Number of patients, *n* *		104	9	95	
Overall medial degeneration, *n*		97 (93.3%)	9 (100%)	88 (92.6%)	>0.99
	Severity, mean (SD)	0.8 (0.4)	0.9 (0.3)	0.8 (0.4)	0.563
Atherosclerosis, *n*		58 (55.8%)	7 (77.8%)	51 (53.7%)	0.293
	Severity, mean (SD)	0.4 (0.5)	0.8 (0.4)	0.3 (0.5)	0.009
Inflammation, *n*		2 (1.9%)	0	2 (2.1%)	>0.99
Severity, mean (SD)		0.1 (0.14)	0	0.02 (0.14)	0.662

SD = standard deviation. * Number of patients who have a histopathology of the aorta.

**Table 4:** Patient outcomes.

Variables	All Patients	With Diabetes	Without Diabetes	*p*-Value
Number of patients, *n*	123	11	112	
Stroke, *n*	38 (30.9%)	5 (45.5%)	33 (29.5%)	0.312
Acute kidney insufficiency, *n*	46 (37.4%)	4 (36.4%)	42 (37.5%)	>0.99
Dialysis	16 (13.0%)	2 (18.2%)	14 (12.5%)	0.635
Respiratory insufficiency, *n*	16 (13.0%)	1 (9.1%)	15 (13.4%)	>0.99
Heart failure, *n*	5 (4.1%)	0	5 (4.5%)	>0.99
MSOF, *n*	23 (18.7%)	2 (18.2%)	21 (18.8%)	>0.99
Early reintervention, *n*	14 (11.4%)	1 (9.1%)	13 (11.6%)	>0.99
Aortic root reintervention, *n*	8 (6.5%)	0	8 (7.1%)	>0.99
Ascending aorta reintervention, *n*	4 (3.3%)	0	4 (3.6%)	>0.99
Descending aorta intervention	4 (3.3%)	0	4 (3.6%)	>0.99
Intraoperative mortality, *n*	17 (13.8%)	1 (9.1%)	16 (14.3%)	>0.99

SD = standard deviation; MSOF = multisystem organ failure.

**Table 5:** Cox multivariable proportional hazards regression analysis of perioperative variables potentially associated with all-cause mortality among patients with and without diabetes.

Variable	Unadjusted HR (95% CI)	*p*-Value	Adjusted HR (95% CI)	*p*-Value
Diabetes vs. no	1.55 (0.73–3.28)	0.253	0.69 (0.21–2.23)	0.537
Male vs. female	0.93 (0.55–1.59)	0.806	1.01 (0.50–2.05)	0.981
Age, y	1.02 (1.00–1.04)	0.120	1.05 (1.01–1.09)	0.019
Hypertension	1.04 (0.58–1.85)	0.902	0.77 (0.34–1.73)	0.530
Smoking	0.65 (0.29–1.43)	0.279	0.75 (0.27–2.10)	0.582
Shock	1.81 (1.07–3.07)	0.028	0.76 (0.29–2.00)	0.577
Tamponade	1.73 (1.03–2.92)	0.036	0.89 (0.39–2.03)	0.781
Ascending aorta tear	1.06 (0.63–1.79)	0.285	0.86 (0.41–1.81)	0.699
Previous cardiac surgery	0.63 (0.25–1.58)	0.219	0.71 (0.19–2.61)	0.601
Visceral malperfusion	1.36 (0.58–3.18)	0.477	2.19 (0.63–7.60)	0.218
Preoperative lactate	1.19 (1.12–1.27)	<0.001	1.31 (1.14–1.50)	<0.001
Bicuspid aortic valve	0.57 (0.18–1.82)	0.343	2.40 (0.67–8.55)	0.178
Atherosclerosis	0.88 (0.48–1.62)	0.679	0.91 (0.47–1.75)	0.767

HR, hazard ratio; CI, confidence interval; y = years.

## Data Availability

All authors had full access to all of the data in this study and take responsibility for the integrity of the data and the accuracy of the data analysis. The data underlying this article will be shared upon reasonable request to the corresponding author.

## Abbreviations

The following abbreviations are used in the manuscript:

ATAAD	acute type A aortic dissection
CT	computer tomography
SD	standard deviation
STJ	sinotubular junction
